# Against Moral Panic and Citation Fiction: A Critique of “Panem, Corticoids and Circenses” and a Proposal for Editorial Gatekeeping on Reference Integrity

**DOI:** 10.1111/bioe.70099

**Published:** 2026-03-19

**Authors:** Ognjen Arandjelović

**Affiliations:** ^1^ University of St Andrews, North Haugh St Andrews UK

**Keywords:** autonomy, citations, doping, editorial policy, enhancement, harm reduction, non‐maleficence, scholarly integrity

## Abstract

The proposed Enhanced Games have become a convenient stage for bioethical sermonising about risk, authenticity, and the “spirit of sport”. This is epitomized by a recent article arguing that institutionalizing pharmacological enhancement under the “pretence of medical supervision and personal autonomy” would redefine human excellence in “purely biochemical terms”, erode ethical norms, normalize doping, and burden public health systems. The present paper offers a two‐level response. First, I criticise the argument on its merits. Its central claims rely on rhetorical inflation, false dichotomies, and circular appeals to unspecified ethical norms. The risk argument is selectively paternalistic, treating pharmacological risk as uniquely disqualifying while ignoring the substantial and well‐documented risks that elite and even mass‐participation sport already normalizes. The treatment of autonomy is reduced to assertions rather than argument; the depiction of medical supervision as mere “pretence” is offered without evidence, despite public‐facing claims by Enhanced Games proponents that they seek clinically supervised enhancement. Second, and more seriously, I turn my attention to an audit of the target article's scholarship. Multiple citations in Demas's article appear to be bibliographic fictions presented as high‐profile clinical evidence on performance‐enhancing drugs. Even when genuine sources are cited, they are invoked for conclusions that those sources do not sustain. This is a serious breakdown of the minimal contract between author, reviewer, and reader, one that demands that cited evidence exists and supports the claims made.

## 1 Introduction

The Enhanced Games present themselves as an “alternative” sporting event in which performance enhancement is permitted under a model described by proponents as “safe, responsible, and clinically supervised” [1]. This sharply contrasts Demas's recent commentary “Panem, corticoids and circenses: the ethical fallout of Enhanced Games” which is representative of a popular stance concerning performance‐enhancing drugs (PEDs) [2]. For this reason, the commentary is useful as a representative example through which I wish to address the posture in general. Demas frames the Enhanced Games as a paradigmatic moral hazard, one “institutionalizing” performance‐enhancing drug use “under the guise of autonomy and medical supervision”, promoting “a profound redefinition of athletic values”, and threatening a “gradual erosion of ethical norms”.

I share one of Demas's background assumptions, namely that unrestricted, unsupervised enhancement can impose serious risks. Anabolic‐androgenic steroids (AAS) in particular are associated with adverse physiological effects (e.g., unfavourable changes in lipid profiles are among the more consistently supported findings in the human literature [3]). Certain classes of androgenic steroids (notably many 17α‐alkylated oral compounds) are implicated in clinically significant liver injury [4, 5]. These are real concerns.

But there are two ways to reason from such concerns. One is careful, seeking to specify what the risks are, how likely they are under realistic usage patterns, what mitigations exist, what coercive dynamics may arise, and what regulatory architecture best reduces total harm while respecting adult autonomy. The other involves moral panic, inflating, rashly generalizing, and treating pharmacology as a uniquely corrupting force that uniquely transforms familiar trade‐offs in sport into unprecedented ethical catastrophe.

In this paper, I respond in a deliberately uncompromising way but without treating rhetoric as evidence. In medical ethics, moral language can be illuminating but it can also function as a substitute for argument. The latter is not acceptable. My thesis is that Demas fails on two fronts:

1. Ethical argumentation: key conclusions are asserted rather than defended, with the text repeatedly sliding from contested premises (about autonomy, consent, and harm) to emphatic prescriptions and warnings.
2. Scholarly reliability: several central empirical claims are supported by references that appear not to exist, while others do not support the claims for which they are cited.

The second point is not highfalutin pedantry. In ethical debates, honest disagreement is common and unproblematic, but when evidential support is unreliable, the disagreement ceases to be primarily normative, becoming epistemically compromised. Moreover, academic journals must play a central role here; if basic reference accuracy is not enforced, the entire genre of “rapid commentary” can become a vehicle for unsupported claims shielded by perceived expertise.

The main contribution of the paper therefore comes in two parts. I first address the substantive ethical claims (risk, autonomy, non‐maleficence, and the alleged “erosion” of norms). I then turn to the distinct but related question of scholarly reliability and publication ethics, using this case as a concrete illustration, and conclude with complementary proposals for procedural improvement.

## 2 Materials & Methods

### 2.1 Normative Claims, Empirical Claims, and Rhetorical Cues

Demas's central argument can be summarized as follows (I quote key phrases to preserve the original rhetoric which is of importance here; all emphasis is mine):

- The Enhanced Games institutionalize enhancement under the “*pretence* of medical supervision and personal autonomy” [2].
- The Games' ethos embodies a “redefinition of human excellence in *purely biochemical terms*” [2].
- “Long‐term medical risks are well documented” [2], and the normalization of such risk will burden public health systems.
- The broader societal result is “a gradual erosion of ethical norms” and a “reinforcement of the idea that performance is only valuable when amplified by science” [2].
- These practices will become “normalised, institutionalised and eventually expected” [2].

The key problem with this line of argumentation lies in its conflation of normative claims (what should be done, what is unethical, what “erosion” is), empirical claims (what harms are documented, what practices will spread, what pressures will arise), and rhetorical cues (“pretence”, “guise”, “dystopian”, “commodified”, “spectacle”). This conflation is instrumental because the paper relies on the reader's affective response. If an author uses terms like “pretence” and “guise”, the burden is to *show* deception, not merely to insinuate it. Similarly, the claim that “ethical norms are undermined” can either be an argument (with identified norms and causal pathways) or an emotive hook that simply restates disapproval. The paper's general approach is thus: (i) assert that Enhanced Games' framing of autonomy and supervision is a sham, (ii) assert a cultural‐moral degradation thesis, (iii) bolster the health‐risk premise by appeal to “well documented” evidence, and (iv) infer a policy‐oriented alarm of needing urgent multidisciplinary intervention.

My critique that follows is correspondingly structured, showing, first, why the moral conclusions do not follow, and second, why the health‐evidence presentation is not merely weak but, in several cases, not real.

### 2.2 Reference Verification Procedure

In all cases of citations herein described as invented, the following check was conducted. First, Google Scholar (https://scholar.google.com/) was searched for the exact paper title. All searches failed to find a match. Second, a relaxed search was conducted in an attempt to find similarly titled articles whose titles may have been mistyped. All searches failed to find a match. Third, a Google (https://www.google.com/) search for the exact title was performed. In all cases, Demas's article yielded the sole hit. PubMed and Crossref/DOI lookup was performed next, again in all cases producing a negative result. Lastly, the journal web site was used to search for the article title (which universally failed to find a match), and then to check what content, if any, is located at the exact bibliographic coordinates (volume, number, pages) specified. Again, in all cases the content was found not to match Demas's citation.

## 3 Results

### 3.1 Substantive Critique

#### 3.1.1 Setting the Stage

Early in the commentary Demas writes that the Enhanced Games will allow PED use “under the pretence of medical supervision and personal autonomy” [2]. To call something a *pretence* is an allegation of misrepresentation, not a morally neutral description of a self‐evident fact. Yet, no evidence is offered either that the claims of medical supervision are knowingly false, or that the claims of participant autonomy are merely theatrical. One could plausibly argue that “medical supervision” offered by the Enhanced Games organizers is inadequate, in principle or in practice. One could also plausibly argue that athletic autonomy is compromised by incentives and coercive pressures. But these are arguments which as such require specifics and, given their empirical nature, *evidence*. Merely declaring “pretence” performs a moral verdict while bypassing its justification.

This early rhetorical move sets the scene for the entire narrative that follows by creating an asymmetry whereby the reader is invited to treat the organizers' claims as being in bad faith by default. By adding that the games are “backed by substantial private investments”—which in and of itself has no dialectic power in the argument as stated—the effect is amplified by triggering further affective response from the reader. That is moralization rather than ethical analysis. Worse, it biases subsequent discussion about consent, harm reduction, and professional ethics, because the conclusion (that supervision and autonomy are sham) is already built into the framing.

#### 3.1.2 Category Error by Adjective

Demas claims that the Enhanced Games point toward “a broader ideological shift: the redefinition of human excellence in purely biochemical terms” [2]. Therefrom two problems arise immediately.

First, it is difficult to be charitable regarding Demas's qualification by “purely”. Sport has never been “purely” anything. Elite performance is *already* a hybrid product of genetics, training, coaching, technology, medicine, psychology, nutrition, and institutional support. Modern sport is saturated with biochemical interventions that are routine and socially accepted, such as ingestion of caffeine and creatine, electrolyte manipulation strategies, carbohydrate periodization, use of altitude tents, and so on [6]. If by “biochemical” Demas means “biochemical means of affect physiology”, then sport was biochemical long before any PED controversy. If ‘biochemical’ is instead a coded way of saying something much narrower, such as “using pharmacology”, then Demas is importing a conclusion that requires defence, namely that pharmacological enhancement is not merely *one* contributor among others but a redefinition of excellence.

Second, once moral panic is replaced with conceptual clarity, a continuum emerges, revealing that PEDs differ one from another substantially by risk profile, reversibility, dose‐response, interaction effects, and monitoring requirements. Treating them as a single category is intellectually lazy and medically misleading. Even within anabolic‐androgenic steroids (AAS), risk profiles vary substantially by compound, dose, route, duration, and user characteristics [7]. An ethical analysis that flattens this heterogeneity is careless.

#### 3.1.3 Medical Risks

Demas states that “the long‐term medical risks are well documented” and then gestures at “an extensive body of literature” describing cardiovascular, hepatic, metabolic, and neuropsychiatric harms [2].

The general idea that PED use can have serious health consequences is not controversial. The Endocrine Society scientific statement is explicit that adverse effects are underappreciated and that the evidence base is uneven, particularly because many users are not elite athletes and because illicit use complicates study designs [7]. Despite this, Demas's “well documented” premises amplifier falls flat because four out of six citations offered are invented.^1^ Of the remaining two, one is outdated, being over 40 years old, and the other does not support Demas's claim that “the long‐term medical risks are well documented”, instead stating:

*The only physical complication of AAS use that receives definitive support from such investigations is unfavourable changes in blood lipid profiles*.

and

*Epidemiological attempts to determine whether AAS use triggers violent behaviour have failed, primarily because of high rates of non‐participation*.

Lest I be misunderstood, by highlighting these problems I do not mean to allege intent. Such allegation requires evidence. Rather, I restrict myself to the claim of reliability failure. To all this I shall come back shortly; for now, I proceed with the argument concerning the substance of the commentary.

The deeper philosophical mistake of interest here is the misuse of risk as a conversation‐stopper. For the sake of argument, let us grant the substantive point that PED use can be risky. Does that actually entail Demas's moral conclusions? Certainly not automatically. Risk is a *parameter* in ethical analysis, not a verdict. Sport already involves systematic risk‐taking, including amongst others overuse injuries, concussions, eating disorders, and endocrinological disruption from relative energy deficiency [8, 9]. Marathon running debates illustrate the same point, namely that risk is real but that so is the reward [10]. In short, the distribution and magnitude of risk matter, as do, above all else, the sportsperson's values and preferences that dictate how these are balanced.

If risk alone justified sweeping prohibitionist conclusions, a large fraction of competitive sport would be ethically suspect. What matters here are comparative, governance‐based questions about baselines, monitoring, externalities, and the conditions for informed and voluntary choice.

#### 3.1.4 Non‐Maleficence vs Supervised Risk

Demas writes:

*Medical professionals may find themselves in ethically untenable positions: should they refuse to supervise these enhancements and risk marginalization, or participate and compromise the principle of non‐maleficence?*

Demas [2]

This is one of the most revealing lines in the piece because it presupposes what it needs to argue, namely that participation in supervision *compromises* non‐maleficence.

Non‐maleficence in clinical ethics is not a simplistic ban on all risk‐imposing intervention. Medicine routinely facilitates risk‐bearing projects when they are competently informed and aligned with patient values, such as elective surgery, fertility treatments with non‐trivial risks, high‐risk obstetrics decisions, palliative sedation trade‐offs, etc. The ethical work is typically done by (i) informed consent, (ii) proportionality, (iii) competence and voluntariness, and (iv) mitigation and monitoring [11]. These are intra‐clinical standards that govern how risk may be managed within medical practice. They do not imply a general harm‐reduction licence, since clinical permissibility itself is constrained by extra‐clinical side‐constraints on what kinds of acts medicine may properly facilitate at all, irrespective of whether doing so might reduce harm.

If an athlete autonomously values certain performance ideals and is willing to accept specific medically characterized risks, the ethical question is not simply one of risk vs. no risk. Rather, the central questions concern the nature and magnitude of the risk, the relevant constraints, the monitoring arrangements, and the protections against coercion and exploitation. That is precisely where supervision can *serve* non‐maleficence by reducing expected harm relative to clandestine, poorly monitored use. The harm‐reduction logic is not unusual in the doping debate; indeed, Kayser et al. [12] explicitly argue that an unattainable “doping‐free” ideal invites hypocrisy and that controlled approaches aimed at harm reduction may be more viable.

If Demas wants to claim that supervision compromises non‐maleficence, he needs to articulate a principle stronger than a ban on participation in risky activity. Such argument could, for example, go in one of the following directions:

- Enhancement is categorically outside the clinician's proper role.
- Consent in elite sport is necessarily invalid.
- The expected harms are so severe that no athlete could rationally endorse them (thus nominal “consent’ implies manipulation or coercion).
- Social externalities are so large that individual consent cannot license them.

But Demas does not supply such an argument. Instead, the dilemma is turned into a rhetorical performance that portrays supervision as moral contamination.

#### 3.1.5 Social Values, Performance, and Persons

Similar dialectic weaknesses persist throughout the commentary. Thus, Demas further writes that the controversy challenges us to consider “whether we value performance more than persons” [2].

While rhetorically forceful, this framing latently sneaks in a veiled, undemonstrated inference. In particular, it treats enhancement as evidence that persons are being subordinated to performance. Yet, in many athletic contexts the motivational structure is the opposite; athletes value the practice, the craft, the embodied achievement, and the experience of self‐transcendence within the sport. When the ability to compete ends—and in many sports it does so at a very young age—the loss can be profound. The desire to extend performance ceilings can be an expression of an athlete's values, quite the opposite of a betrayal of them. For example, an analysis of bodybuilders' “vocabularies of motive” found that users justified AAS through self‐fulfilment narratives, framing it as a rational means to personal ends like bodily mastery. Respondents emphasised the journey to build something extraordinary from oneself, focusing on internal rewards like a stronger sense of self, rather than winning or beating others.

The point that Demas never seriously entertains is that PED use can be construed as a form of taking athletes' preferences seriously rather than imposing paternalistic ideals of what they should value. On that view, the question is not of performance vs. persons, but of *whose values* are governing the athlete's body, those of the athlete or those of an external moral regime that claims to know better.

I should not be misunderstood as offering a free pass for anything goes attitude. Not at all. The autonomy story *can* be compromised by coercive incentives, misinformation, or economic desperation, but this is nothing uniquely applicable to PED use by athletes. My point is rather that the rhetoric of “performance vs. persons” pre‐judges the conclusion by assuming that enhancement is necessarily a form of devaluation. That position is one that must be argued, not merely assumed by leaning on the readers' affective response.

#### 3.1.6 Erosion of Ethical Norms

The next example features what should by now be a familiar argumentation error. Demas draws the following conclusion: “The result is a gradual erosion of ethical norms” [2].

This line too exemplifies circular reasoning, moral reasoning specifically, by implying that ethical norms are those that condemn the relevant behaviour, so the behaviour erodes ethical norms by definition. That is definitional gatekeeping rather than analysis. If one wants to argue that PED normalization erodes *specific* norms, these must be named and defended which Demas neither attempts nor acknowledges. For completeness, I note that candidates for such norms might include fairness in competitive classification, non‐exploitation, protection of minors, or honesty about what audiences are watching. But even then, the direction of argument is not obvious. Some norms may be better served by transparency and pluralism than by moral imposition and policing.

#### 3.1.7 Normalization, Institutionalization, Expectation

In the last example that I address directly here, Demas warns that “under the guise of control and supervision, these practices risk becoming normalised, institutionalised and eventually expected” [2]. There are at least three gaps in this.

##### 3.1.7.1 Neutrality of Normalization

To say something is “normalized” is to describe a social fact, not to condemn it. If normalization is wrong, the author must say why. Without this, “normalized” functions as a trigger‐word, deriving its argumentative force from leaning on the term's usually negative connotations.

##### 3.1.7.2 Positive Institutionalization

Institutionalization includes the existence of rules, monitoring, constrained formularies, age limits, disclosure regimes, adverse‐event reporting, independent oversight. These are not in and of themselves ethical disasters; quite in fact, they can be the exact opposite. Indeed, they are the common, accepted, and useful tools by which societies reduce harms associated with high‐risk practices.

##### 3.1.7.3 Pluralism

Even if some sports settings gravitate toward enhancement, it does not follow that enhancement necessarily becomes universally expected. Real‐world sport already accommodates pluralism with separate federations and divisions having different rules and aesthetics. Bodybuilding provides an illustrative example. It is a sport that stands above all others in terms of prevalence of PED use; WADA's Anti‐Doping Testing Figures make this point sharply.^2^ Moreover, drug use within the relevant community is normalized. Importantly, since competitive bodybuilding continues to be a relatively niche sport, its independence from general sporting organizations such as the IOC, allowed it to develop its own character. In countries such as the UK where PED use itself is not against the law, there is an entire ecosystem of different organizations, some of which prohibit lifetime PED use (e.g., the UK National Physique Association), some of which allow PED use (e.g., the United Kingdom Bodybuilding and Fitness Federation), and some which implement time‐limited prohibition (e.g., the British Natural Bodybuilding Federation). These offer genuine choice to athletes and promote mutual respect: as everybody competes on a level playing field *and* has a choice which field that is, PED‐using and so‐called “natural” athletes often express nothing but admiration for one another, being united by a common passion despite diverging in their disposition to risk.

In short, the policy‐relevant question is what institutional design best preserves meaningful choice, avoids coercion, and protects vulnerable participants. Blanket moral condemnation does not answer that.

### 3.2 Reference Integrity Findings

As noted in the introduction, in this, second part of the present paper, I turn my attention to an issue entirely orthogonal to that discussed thus far: one that concerns academic integrity and culture. The most serious problem with Demas's commentary is not that it is opposed to Enhanced Games on poorly argued grounds but rather that it presents itself as evidentially supported while relying on references that appear invented (see Appendix A). COPE treats falsified references as serious misconduct [13]; the ICMJE has recently emphasised author responsibility for the accuracy of cited references [14].

These standards are minimal. A journal publishing a commentary with multiple false citations, and seemingly nobody noticing this—not the reviewers, not the editors, not the readers—highlights a serious problem. This is especially worrying in medical ethics, where commentary can shape public and professional perceptions quickly, and where ethics language can launder factual unreliability.

## 4 Discussion

It is tempting to treat reference failures as a mechanistic problem that could be addressed through technology and bureaucracy by installing more automated checks, tightening and enforcing checklists, reminding authors of their obligations. Not only banal, that response misdiagnoses the failure mode. In a publication culture where fast‐turnaround commentaries are rewarded, reviewers often treat references as decorative rather than load‐bearing, and journals rarely suffer meaningful consequences for bibliographic unreliability, low‐grade fabrication, and high‐grade sloppiness become rational strategies.

The core issue, then, is *not* attempting to help authors avoid mistakes. Authors know this. Rather, the challenge is in making it *costly* (in probability and consequence) to submit work with citations that do not exist or do not support what they are cited for. In other words, what is needed is deterrence, not assistance; accountability, not coaching. I elaborate next.

### 4.1 Validation as Remedy

Routine automated validation can catch accidental errors, but it also risks functioning as an *anti‐detection tutor*, an interactive system that teaches negligent or opportunistic authors how to edit their way past superficial screening. In that sense, a purely front‐end, author‐visible validator is analogous to a weak plagiarism checker, improving quality only insofar as it also improves the craft of evasion.

If automation is to be used, to ensure alignment with the desired ethos of academic publishing, it should be *non‐coaching*, that is, it should not operate as a friendly, iterative feedback loop. Its function should rather be to create an audit trail for editors and reviewers, and to inform post‐acceptance checks and sanctions. The general aim is to send a clear message that references serve a serious epistemic and governance purpose.

### 4.2 Deterrence

As already noted, COPE guidance treats falsified references as a serious integrity issue [13]. Yet, the perceived risk of being caught is often low, especially in short ethics pieces that trade in general claims (“well documented”) rather than in tight empirical inferences. Journals can change this incentive landscape without pretending that authors “just need reminders”. An improved deterrence approach could comprise the following three components.

#### 4.2.1 Adversarial Spot‐Checks After Provisional Acceptance

Instead of (or in addition to) pre‐submission coaching checks, journals could perform a small number of editorial spot‐checks *after* a manuscript is conditionally accepted. Selection could be partially random and partially risk‐based (e.g., assessed based on unusually authoritative empirical rhetoric, disproportionately long reference list, claims that would be reputationally damaging if unsupported). These checks would focus on two questions only, namely whether the cited item exists as specified, and whether it, on a reasonable reading, supports the claim for which it is cited. The overarching aim is to raise the expected cost of submission with invented citations.

#### 4.2.2 Consequence Clarity

ICMJE already emphasises author responsibility for the accuracy of cited references [14]. Journals should make consequences explicit in author guidelines and then ensure that they are applied. Not every error warrants the same response, but *non‐existent* citations must not be treated like a typesetting problem. At minimum, measures along the following lines should be implemented:

- Pre‐publication: authors are required to supply verifiable metadata for flagged references.
- Post‐publication: corrections are issued rapidly and prominently; where multiple fabricated or grossly misleading citations are identified, a formal review is initiated, in sufficiently serious cases leading to retraction.
- Repeat behaviour: escalating sanctions (e.g., temporary submission bans) are instituted and applied transparently.

Punition should not be a nominal, box‐ticking exercise but part of a serious quality‐control mechanism.

#### 4.2.3 Plausible Deniability Removal

Fabricated or careless citations are hardest to detect when broad empirical claims are supported by long, non‐specific reference lists, or when the claims in question sound familiar and therefore attract less scrutiny than they deserve. Journals can reduce plausible deniability by requiring a brief evidential note, submitted separately from the main manuscript, explaining how the cited sources support a small number of key, empirically loaded assertions (such as those seen earlier, e.g., “long‐term risks are well documented”). This forces authors to commit, gives reviewers a tractable checking task, and reduces the practice of citations to prestigious journals being used as a disarming proxy for epistemic rigour.

### 4.3 Accountability

Reference integrity improves when failures are *socially salient*. The literature on reference accuracy shows that errors are common across biomedical fields [15] and the key question is whether journals treat them as understandable, tolerable errors or as breaches of scholarly trust. A journal that is serious about integrity should:

- Publish corrections in a way that is indexed and prominent.
- Maintain a consistent editorial stance that citation existence and traceability of cited sources is a serious matter.
- Treat medical ethics commentaries in particular as high‐risk vehicles for the spread of unsupported claims, because they are often rhetorically forceful while subject to lighter peer scrutiny than full research articles.

Such measures would improve citation accuracy directly and, just as importantly, signal that the journal takes reference integrity seriously.

### 4.4 Genre‐Specific Reform

The deeper problem may be that the current “rapid commentary” genre rewards overconfidence while being insufficiently demanding of evidential and argumentative support. The genre is valuable, filling a niche within the publishing landscape that other article types cannot. However, its epistemic standards would benefit from being tightened. A simple editorial norm would help; when a commentary makes strong empirical claims (“well documented”, “extensive literature”, “clear evidence”), it should either cite a small number of authoritative syntheses (e.g., major scientific statements), or downgrade its language to match what it can actually support. In the present case, the demand is ethical, since the critique is premised on claims about health risks and therefore requires an evidential chain that exists and can be checked.

In short, the solution is not to give authors better proofreading tools but to alter the incentive structure so that fabricated or careless referencing becomes a losing strategy.

## 5 Conclusion

The ethical debate about enhancement, PEDs, and new sporting formats is worth having. However, positions within that debate must be argued rather than smuggled under the cover of emotive rhetoric, and empirical evidence provided rather than invented.

Demas [2] leans heavily on charged phrasing (“pretence”, “purely biochemical”, “dystopian”, “erosion”) where argument is required, and it frames a purported clinical dilemma as if medical supervision necessarily violates non‐maleficence—a claim that is, at minimum, question‐begging. The strongest objection to the Enhanced Games programme does not lie in “purity” or “biochemical excellence” but in the coercion, arms‐race dynamics, and inequality of access.

More decisively, the paper's scholarly apparatus appears compromised by multiple citations to non‐existent references and work that does not support the claims made. In an academic journal, especially a medical ethics journal, that combination should be unacceptable.

Both parts of the present paper, those concerning Demas's argument and the quality of scholarship on which it relies, point to the same core lesson. The key problem is institutional, and for journals to play their vital role in facilitating ethical challenge and debate, they must ensure appropriate mechanisms are in place to keep what they publish tied to reality.

## Conflicts of Interest

The author declares no conflicts of interest.

## Endnotes

1 See Section 2.2 for the reference verification procedure and search protocol.

2
https://www.wada-ama.org/sites/default/files/resources/files/2017_anti-doping_testing_figures_en_0.pdf.

## Appendix

**Table:**

Citation as listed by Demas	Problem	What occupies the cited coordinates (evidence)
Smith J, Doe A, Brown B, et al. Cardiovascular Effects of Anabolic Steroid Use in Athletes: A Prospective Cohort Study. *N Engl J Med* 2015;372:365–74.	The title does not match the indexed paper at that volume and page range; no indexed record matching this citation was found on PubMed, Crossref, Google Scholar, and the journal site.	The article closest to the specified bibliographic coordinates is a case study of 60‐year‐old woman with abdominal pain, dyspnea, and diplopia [16].
Lee H, Kim Y, Patel R, et al. Doping in Elite Sports: A Global Perspective on Health and Regulation. *Lancet* 2019;394:2108–16.	The title does not match the indexed paper at that volume and page range; no indexed record matching this citation was found on PubMed, Crossref, Google Scholar, and the journal site.	The article closest to the specified bibliographic coordinates is a trial on efficacy and safety of upadacitinib in patients with ankylosing spondylitis [17].
Johnson R, Miller S, Davis P, et al. Long‐Term Health Outcomes in Athletes Using Performance‐Enhancing Drugs. *JAMA* 2018;320:1452–60.	The title does not match the indexed paper at that volume and page range; no indexed record matching this citation was found on PubMed, Crossref, Google Scholar, and the journal site.	The article closest to the specified bibliographic coordinates is a randomized trial on immediate vs delayed pushing in labour [18].
Kumar P, Singh A, Johnson K, et al. Neurocognitive and Behavioral Consequences of Anabolic Steroid Use in Professional Athletes. *J Neurol Neurosurg Psychiatry* 2020;91:1085–92.	The title does not match the indexed paper at that volume and page range; no indexed record matching this citation was found on PubMed, Crossref, Google Scholar, and the journal site.	The article closest to the specified bibliographic coordinates is a review on spinal and bulbar muscular atrophy [19].
Ashenden MJ, Schumacher YO, Sharpe K. Contemporary Issues in Doping Control: Collusion and Concerns in Anti‐Doping. *Sports Med* 2011;41:881–4.	The title does not match the indexed paper at that volume and page range; no indexed record matching this citation was found on PubMed, Crossref, Google Scholar, and the journal site.	The articles closest to the specified bibliographic coordinates concern rib stress fractures among rowers and physiological and nutritional aspects of post‐exercise recovery [20, 21].
